# Nitrogen application and different water regimes at booting stage improved yield and 2-acetyl-1-pyrroline (2AP) formation in fragrant rice

**DOI:** 10.1186/s12284-019-0328-4

**Published:** 2019-10-03

**Authors:** Zhaowen Mo, Yanhong Li, Jun Nie, Longxin He, Shenggang Pan, Meiyang Duan, Hua Tian, Lizhong Xiao, Keyou Zhong, Xiangru Tang

**Affiliations:** 1 Department of Crop Science and Technology, College of Agriculture, South China Agricultural University, Guangzhou, 510642 Guangdong China; 2 Scientific Observing and Experimental Station of Crop Cultivation in South China, Ministry of Agriculture.P. R. China, Guangzhou, 510642 China; 3 Agro-innovative Demonstration Base Guangdong Academy of Agricultural Sciences, Guangzhou, 510642 China

**Keywords:** 2-acetyl-1-pyrroline, Biochemical parameters, Fragrant rice, Nitrogen, Water, Yield

## Abstract

**Background:**

Water (W) and nitrogen (N) management generally cause regulations in the 2-acetyl-1-pyrroline (2AP) accumulation in fragrant rice; nevertheless, the feasibility of such management strategies at booting stage in improving 2AP accumulation has not been examined in details.

**Methods:**

Field experiments were conducted in the early season (March–July) and repeated in the late season (July–November) in 2013. The treatments were applied urea (90 kg ha^− 1^), calcium super phosphate (90 kg ha^− 1^) and potassium chloride (195 kg ha^− 1^) as basal fertilizer, and urea (65 kg ha^− 1^) at tillering stage. Three N levels i.e., 0 kg N ha^− 1^ (N1), 30 kg N ha^− 1^ (N2), and 60 kg N ha^− 1^ (N3) and three water levels i.e., W1 treatment (well-watered treatment with water layer of 2–4 cm), W2 treatment (soil water potential was − 15 ± 5 kPa), and W3 treatment (soil water potential was − 25 ± 5 kPa) at booting stage was set up for three rice varieties i.e., Nongxiang 18, Yungengyou 14 and Basmati. The grain yield, head milled rice yield, 2AP contents and the biochemical parameters related to 2AP formation were investigated.

**Results:**

Result indicated that W and N dynamics regulated the grain yield, head milled rice yield, and 2AP contents in brown rice across three varieties. The N2 and N3 treatment significantly increased the 2AP contents in brown rice by 9.54% and 11.95%, and 8.88% and 32.54% in the early and the late season, respectively; improved grain yield and head milled rice yield. The W3 treatment improved grain yield, head milled rice yield and 2AP content. Significant W and N interaction effect on 2AP content in brown rice was detected, where the W3 N3 treatment showed the strongest interaction regarding improvement of 2AP contents in brown rice. The 2AP accumulation and its related biochemical parameters and their relationships in different plant tissues at different growth stages under W and N treatments had also been assessed. The 2AP content, P5C content and DAO activity during grain filling periods was highly related to the 2AP content in brown rice.

**Conclusion:**

This study revealed that the 60 kg N ha^− 1^ coupled with − 25 ± 5 kPa treatment showed the best positive effects on yield and aroma in fragrant rice, suggested that water and nitrogen management at booting stage can improve grain yield and fragrance in fragrant rice. However, further study to evaluate the metabolic and molecular basis of 2AP accumulation in fragrant rice is needed.

**Electronic supplementary material:**

The online version of this article (10.1186/s12284-019-0328-4) contains supplementary material, which is available to authorized users.

## Introduction

Fragrant rice such as the Pakistani and Indian ‘Basmati’ and Thai ‘Jasmine’ are worldwide famous fragrant rice types among consumers with high trade prices (Sakthivel et al., 2009). Studies reported that the fragrant rice can volatilize special fragrant compound which distinguish it from the non-fragrant rice (Bryant and McClung, 2011; Wakte et al., 2017; Routray and Rayaguru, 2017; Wei et al., 2017). Many volatile compounds have been detected in fragrant rice, but the 2-acetyl-1-pyrroline (2AP) is considered to be the most important one (Buttery et al., 1983; Buttery et al., 1988; Champagne, 2008; Jezussek et al., 2002). Generally, the 2AP could be detected only in above ground plant parts of fragrant rice such as grain, stem sheath and leaf (Buttery et al., 1983; Maraval et al., 2010).

In general, the genetic factor is recognized to play the important role in affecting the aroma biosynthesis in fragrant rice (Lorieux et al., 1996; Bradbury et al., 2008; Fitzgerald et al., 2008), however, many environmental factors and management practices have been reported to affect the 2AP biosynthesis in fragrant rice (Bhattacharjee et al. 2002; Champagne, 2008; Gay et al., 2010; Yang et al. 2012; Mo et al., 2016, b). For instance, light intensity, temperature, soil nutrient, salinity as well plant nutrition such as silicon, Mn, and Zn application are external plant factors that substantially affect the 2AP contents in fragrant rice (Fitzgerald et al., 2010; Poonlaphdecha et al. 2012; Yang et al. 2014; Li et al., 2016, b; Mo et al. 2015, 2016,b, 2017, 2018, 2019).

Moreover, improvements in 2AP content in aromatic rice grains due to irrigation management were also reported previously (Tian et al., 2010; Tian et al., 2014; Wang et al. 2013). Yoshihashi et al. (2002) reported that mild drought treatment during milking period of fragrant rice increased 2AP content, whereas irrigation dynamics especially shallow-water irrigations/alternate wetting and drying conditions could improve the 2AP contents in grain (Tian et al., 2010; Wang et al., 2013; Bao et al., 2018). Thus, it is feasible to increased 2AP content in fragrant rice with moderate irrigation.

Additionally, N application affected 2AP formation in fragrant rice (Zhong and Tang, 2014). For example, Li et al. (2014, b) reported that water-nitrogen interaction at tillering stage had a significant influence on 2AP content in brown rice. Ren et al. (2017) reported that application of N at 60 kg hm^− 2^ with less water irrigation at tillering stage could remarkably increase 2AP content in grain, whilst, Mo et al. (2018) indicated that different N application levels affected the 2AP content in fragrant rice. The N effect on 2AP contents in fragrant rice have also been supported by many previous studies (Itani et al., 2004; Mo et al., 2018, 2019). Therefore, moderate and timely N application is important to improve 2AP content in fragrant rice.

The biochemical parameters involved in the 2AP biosynthesis in fragrant rice could be interpreted from previous studies (Huang et al., 2007, 2008; Chen et al., 2008; Bradbury et al., 2008; Sakthivel et al., 2009; Fitzgerald et al., 2009, 2010; Wakte et al., 2011). In brief, the proline, ornithine, glutamic acid, 1-pyrroline-5-carboxylate, pyrrole and 1-pyrroline were detected as the key precursors for 2AP biosynthsis (Seitz et al., 1993; Huang et al., 2008; Daygon et al., 2017). Besides, the enzymes such as proline dehydrogenase (PDH), ^Δ^1-pyrroline-5-carboxylate synthetase (P5CS), and ornithine aminotransferase (OAT) and diamine oxidase (DAO) were suggested to be highly related to 2AP formation (Chen et al., 2008; Bradbury et al., 2008; Sakthivel et al., 2009; Fitzgerald et al., 2009, 2010; Wakte et al., 2011; Kaikavoosi et al., 2015; Ghosh and Roychoudhury, 2018).

Water and N management are sustainable strategies to modulate the 2AP content in fragrant rice, however, the effect of W and N at booting stage on 2AP content and the related biochemical parameters in different plant tissues has not been examined in detail. Therefore, in this study, field experiments were conducted to i) evaluate the effect of water and nitrogen at booting stage on grain yield, head rice yield and 2AP content in three popular fragrant rice varieties; and ii) to investigate the relationships between 2AP content and the biochemical parameters in different plant tissues at different growth stages across different water and nitrogen levels.

## Materials and methods

### Experimental site description

Field experiments were performed in the early season (March–July) and repeated in the late season (July–November) in 2013 in two adjacent fields at the Experimental Farm of College of Agriculture, South China Agricultural University (SCAU) in Guangzhou, Guangdong Province, P.R. China. Guangzhou has a humid subtropical climate (Table 1). The properties of soil collected from the upper 20 cm are shown in Table 2.

**Table 1:** meteorological data of the experimental site

Month	Mar.	Apr.	May.	Jun.	Jul.	Aug.	Sep.	Oct.	Nov.
Temperature (°C)	19.6	20.9	25.6	28	27.9	27.9	27	23.6	19.5
Humidity (%)	80	86	86	81	82	83	79	66	71
Rainfall (mm)	177.1	268.4	302.7	229.1	273.3	396.6	203.7	5.9	40.3
Sunshine hours (h)	86.8	38.8	73.9	167.3	177.3	162.3	176.1	224.4	150.1

**Table 2:** Properties of the experimental field soil

Properties	Early season	Late season
Organic matter (g kg^− 1^)	23.30	25.70
Total N (g kg^− 1^)	1.10	1.40
Total P (g kg^− 1^)	1.10	1.00
Total K (g kg^− 1^)	24.40	17.50
Available N (mg kg^− 1^)	114.30	85.50
Available P (mg kg^− 1^)	61.30	25.10
Available K (g kg^−1^)	127.00	153.20

### Experimental treatments and design

The experimental treatments were comprised of three N levels i.e., 0 kg N ha^− 1^ (N1), 30 kg N ha^− 1^ (N2), and 60 kg N ha^− 1^ (N3); three fragrant rice varieties i.e., Nongxiang 18, Yungengyou 14 and Basmati and three water levels i.e., well-watered treatment with water layer of 2–4 cm (W1), soil water potential was − 15 ± 5 kPa (W2), and soil water potential was −25 ± 5 kPa (W3), the water treatment was conducted according the method of Yang et al. (2007). The experiments were arranged in a split-split plot design, with N levels in main plot and the fragrant rice cultivar in sub-plots whereas the water treatments in sub-subplot. The sub-subplot size was 3 m × 5 m (375 hills plot^− 1^). All the treatments had three replications. The N and W treatments were carried out from R0 to R4 stage as described by Counce et al. (2000), the treatment period for the early season was from 12th of May to 12th of June, for the late season was from 1st of September to 1st of October, 2013. All the treatments received N (90 kg ha^− 1^), P_2_O_5_ (90 kg ha^− 1^) and K_2_O (195 kg ha^− 1^) in the form of urea, calcium super phosphate and potassium chloride at basal, respectively. Then an additional dose of N (30 kg N ha^− 1^) was applied to all the treatment at tillering stage.

The twenty one-day-old seedlings for early rice and seventeen-day-old seedlings for late rice from wet bed nurseries were transplanted at 2 seedlings per hill at 20 cm × 20 cm planting distance on 31st of March and 2nd of August and harvested on 12th of July and 1st of November for early and late seasons, respectively. All crop managements were in accordance with standard cultural practices and the standard chemical products were used to avoid yield loss and quality change. Apart from the treatment period, all the plots were flooded 3 days after transplanting, and a water depth of 2–4 cm was maintained until 7 days before maturity.

### Sampling and measurement

Plant sampling was carried out as the method describing by Poonlaphdecha et al. (2012). The sampling time was during 9:00–11:00 am at full heading stage (FH), 7 days after full heading (7 d AFH), 14 days after full heading (14 d AFH), 21 days after full heading (21 d AFH), and mature stage (MS). Three representative plants from each plot were washed with tap water and then were immediately divided into panicle, leaves, stem-sheath and root. The plant organs were then washed with distilled water. Each part of the plants were mixed and divided into three portions: one portion was stored at ˗20 °C for determination of 2AP (except the root); one portion was frozen with liquid nitrogen and stored at ˗80 °C for determination of the physiological attributes; the rest portion of the samples were used for determination of water content of the different plant parts. At maturity, grain yield was measured from one unit area (1 m^2^) sampling area within each plot, threshed manually, and then sun dried (adjusted to moisture content of ~ 14%), to record the grain yield. The head milled rice rate was measured as described by Mo et al. (2015) and then the head milled rice yield was calculated.

### The 2-acetyl-1-pyrroline (2AP) content

About 5–10 g of the fresh organs sample and the brown rice was weighted for 2AP measurement. The grains and brown rice sample was grinded whilst the leaves and stem-sheath sample was cut into small segment (0.5 cm length). The measurement of the 2AP content was carried out by using the synchronization distillation and extraction method (SDE) combined with GCMS-QP 2010 Plus (Shimadzu Corporation, Japan) method (Huang et al., 2012; Mo et al., 2015).The condition of the GCMS-QP 2010 Plus was: the gas chromatograph equipped with a RTX-5MS (Shimadzu, Japan) silica capillary column (30 m × 0.32 mm × 0.25 μm). High purity helium gas (99.999%, Guangzhou Gases Co., LTD, China) was the carrier gas at a flow rate of 2.0 mL min^− 1^. The temperature of the GC oven was 40 °C, increased at 2 °C min^− 1^ to 65 °C and held at 65 °C for 1 min, and then increased to 220 °C at 10 °C min^− 1^, and held at 220 °C for 10 min. The ion source temperature was 200 °C. Under these conditions, the retention time of 2AP was 7.5 min. 2-AP content was expressed as ug kg^− 1^ dry weight (DW).

### Proline content

The proline content was measured according to the method of Bates et al. (1973). The plant tissue was extracted in 3% sulfosalicylic acid and kept at boiling water for 10 min. The extract was then centrifuged at 4000 rpm for 5 min, and the supernatant was collected for proline determination. Supernatant (2 ml) was mixed with 2 ml glacial acetic and 2 ml ninhydrine reagent and then kept at boiling water for 30 min. After that, the reaction mixture was extracted by 4 ml toluene, and the extract was centrifuged at 4000 rpm for 5 min. The absorbance was recorded at 520 nm. The proline content was expressed as μg g^− 1^ fresh weight (FW).

### 1-pyrroline-5-carboxylic acid content

The determination of 1-pyrroline-5-carboxylic acid (P5C) content was conducted according the method of Wu et al. (2009). The supernatant (0.3 ml) was added to a mixture containing 0.5 ml of 10% trichloroacetic acid and 0.125 mL of 40 mM o-aminobenzaldehyde. The sample was kept at room temperature for 30 min and then centrifuged at 8000 rpm for 10 min. After centrifugation, the absorbance was measured at 440 nm. The P5C content was expressed in μmol g^− 1^ FW.

### Proline dehydrogenase activity

The proline dehydrogenase (PDH) activity was measured according the methods reported by Tateishi et al. (2005) and Ncube et al. (2013). The reaction mixture contained L-proline (15 mM), cytochrome c (0.01 mM), phosphate buffer (100 m M, pH 7.4), 0.5% (v/v) triton X-100, and the enzyme extract (0.1 mL) in a total volume of 0.5 mL was used. The reaction mixture was incubated at 37 °C for 30 min and the reaction was terminated by adding 0.5 ml of 10% trichloroacetic acid (TCA). Then 0.5 ml of 0.5% 2-aminobenzaldehyde in 95% ethanol was added. The mixture was further incubated at 37 °C for 10 min, centrifuged at 8000 rpm for 10 min and the absorbance was recorded at 440 nm. The PDH activity was expressed as μmol g^− 1^ fresh weight (FW).

### Pyrroline-5-carboxylic acid synthetase activity

The pyrroline-5-carboxylic acid synthetase (P5CS) activity was determined by using the methods reported by Hayzer and Leisinger (1980), Zhang et al. (1995) and Sánchez et al. (2002). Briefly, 0.5 mL of reaction mixture (50 mM Tri-HCl pH 7.0, 50 mM glutamate, 20 mM MgCl_2_, 10 mM ATP, 100 mM hydroxamate-HCl) was mixed with 0.5 mL of enzymatic extracts and kept at 37 °C for 5 min, the reaction was then stopped by adding of 0.5 mL of a stop buffer (2.5% of FeCl_3_ plus 6% of trichloracetic acid, dissolved in 100 mL of 2.5 M HCl). The absorbance was recorded at 535 nm. The P5CS activity was expressed as μmol g^− 1^ fresh weight (FW).

### Ornithine aminotransferase activity

The ornithine aminotransferase (OAT) activity was measured by the methods of Grantham and Barrett (1986), Chen et al. (2001) and Umair et al. (2011). The reaction mixture of 1 mL containing 100 mM potassium phosphate buffer (pH 8.0), 50 mM ornithine, 20 mM α-ketoglutarate, 1 mM pridoxal 5-phosphate, and the enzyme extract (0.1 mL) was incubated at 37 °C for 30 min. The reaction was stopped by adding 0.5 mL trichloroacetic acid (10%) and the color was developed by adding 0.5 ml o-aminobenzaldehyde (0.25%) in ethanol (95%). The absorbance was recorded at 440 nm. The ornithine aminotransferase (OAT) activity was expressed as μmol g^− 1^ FW.

### Diamine oxidase activity

The diamine oxidase (DAO) activity was detected by using the methods as described by Jotova et al. (1999), Su et al. (2005), Xing et al. (2007) and Yang et al. (2011). The reaction solutions (3.0 ml) contained 2.5 mL 0.1 M sodium phosphate buffer (pH 6.5), 0.1 mL crude enzyme extracts, 0.1 mL peroxidase (250 U mL^− 1^) and 0.2 mL 4-aminoantipyrine / N, N-dimethylaniline. The reaction was initiated by the addition of 0.1 mL 20 mM Put. The absorbance change at 555 nm was recorded. The diamine oxidase (DAO) activity was expressed as U g^− 1^ FW.

### Statistical analysis

Analysis of variance (ANOVA) was performed by using Statistix version 8 (Analystical, and Tallahassee, Florida, USA), the means of three replications of grain yield, head milled rice yield and 2AP in brown rice were compared using the least significant difference (LSD) test at *P* < 0.05 level. The regression analysis was performed with mean values of the investigated parameters to test the correlation between 2AP and other parameters using Statistix version 8 (Analystical, and Tallahassee, Florida, USA). All regressions were fitted by linear models, the regression coefficients and significance are shown for *P* < 0.05 and *P* < 0.01.

For multivariate analysis, data were imported into the MetaboAnalyst software (http://www.metaboanalyst.ca; Xia et al., 2009). The heatmap for the investigated parameters was established. The PatternsHunter was established for the parameters of interest. Principal component analysis (PCA), Partial least squares - discriminant analysis (PLS-DA) was performed to examine the intrinsic variation in the parameters, and to reduce the dimensionality of the data. A score plot was used to show the similarities and differences among the parameters. In a score plot the data sets exhibiting similarities are clustered together and those that are different are placed further apart. The loadings plot shows the variables responsible for the variation within the parameters, and the correlations among the parameters. The supervised classification and feature selection method’ Random Forest’ was conducted to evaluate the contributions of the parameters. The hierarchical cluster analysis with pearson’s correlation was performed to explore the presence of clustering patterns among the parameters. The expression patterns and a heat map of each variable were categorized using an average linkage hierarchical clustering program. In addition, the partial least squares (PLS) regression was use to evaluate the prediction of 2-acetyl-1-pyrroline content in grains with the investigated parameters conducted with XLSTAT software (Addinsoft, USA) (Funsueb et al., 2016).

## Results

### Grain yield, head milled rice yield and 2AP content

Varieties (V) differed significantly regarding grain yield and 2AP content in brown rice in both seasons whereas significant differences among varieties regarding head milled rice yield was detected for late season only. For early season, Basmati produced the highest grain yield with a mean value of 7.24 t ha^− 1^, while Yungengyou 14 accumulated the highest mean 2AP content (34.193 μg kg^− 1^ DW) (Table 3). For late season, Yungengyou 14 gave the highest mean grain yield (6.27 t ha^− 1^), head milled rice yield (4.13 t ha^− 1^) and 2AP content in brown rice (145.86 μg kg^− 1^ DW) (Table 4). Water (W) showed significant effects on grain yield and head milled rice yield in both seasons, while significant water effect on 2AP content in brown rice was detected for late season only. For early season, compared with W1 treatment, significant increase in the mean value of grain yield, head milled rice yield and 2AP content for W2 treatment was detected by 25.39%, 34.55% and 8.96%, respectively, while W3 treatment significantly increase mean value of grain yield and head milled rice yield by 18.60% and 32.74%, respectively (Table 3). For late season, W2 and W3 treatment significantly enhanced mean grain yield and head milled rice yield as compared to W1 treatment, W3 significantly improved the 2AP content in brown rice (Table 4). In early season, with N application the mean grain yield and 2AP content was in trend of N3 ≈ N2 > N1. Compared with N1 treatment, N3 treatment significantly improved grain yield and 2AP content by 6.72% and 8.86%, respectively, while N2 treatment significantly increased 2AP content by 9.54% in early season (Table 3). For late season, compared with N1, N2 treatment led to significant improvement in head milled rice yield and 2AP content by 16.32% and 9.12%, respectively whilst N3 treatment significantly increased the grain yield and head milled rice yield and 2AP content by 9.52%, 15.61% and 32.54%, respectively (Table 4). The W × N indicated significant effect on 2AP content in brown rice in both seasons, but significant W × N effect on grain yield, head milled rice yield was detected for late season only (Tables 3 and 4). The difference in grain yield, head milled rice yield and 2AP content in brown rice may be related to the variations in humidity and rainfall where higher humidity and heavy rainfall during the rice growth period in early season than in late season as well as higher air temperature during grain filling period in early season than in late season was detected (Table 1).

**Table 3:** Grain yield, head milled rice yield and 2AP content in brown rice of three fragrant rice varieties grown under different water and nitrogen treatments in early season of 2013

Nitrogen	Variety	Water	Grain yield (t ha^−1^)	Head milled rice yield (t ha^− 1^)	2AP content in brown rice (μg kg^− 1^ DW)
N1	Nongxiang 18	W1	5.83	2.99	15.49
		W2	6.64	3.45	17.12
		W3	6.04	3.21	13.34
	Yungengyou14	W1	5.24	3.10	35.26
		W2	5.48	2.65	36.54
		W3	5.28	2.68	26.58
	Basmati	W1	5.93	2.36	15.47
		W2	8.44	3.47	17.12
		W3	7.08	3.13	12.64
		Mean	6.22 b	3.00 a	21.06 b
N2	Nongxiang 18	W1	5.33	2.53	14.68
		W2	6.12	3.61	16.89
		W3	6.55	3.42	21.78
	Yungengyou14	W1	5.74	2.25	36.65
		W2	6.76	3.41	37.50
		W3	5.21	2.60	35.35
	Basmati	W1	6.13	2.21	16.13
		W2	8.88	3.89	14.57
		W3	7.10	3.17	14.11
		Mean	6.42 ab	3.01 a	23.07 a
N3	Nongxiang 18	W1	5.23	2.30	14.91
		W2	6.59	2.76	19.43
		W3	7.71	3.63	18.38
	Yungengyou14	W1	5.06	2.21	26.69
		W2	6.13	3.12	33.90
		W3	7.45	4.47	39.26
	Basmati	W1	5.95	1.95	16.56
		W2	8.22	3.10	15.95
		W3	7.41	2.75	21.27
		Mean	6.64 a	2.92 a	22.93 a
ANOVA		Variety (V)	^b^	ns	^b^
		Water (W)	^b^	^b^	ns
		Nitrogen (N)	^a^	ns	ns
		V × W	^b^	^b^	ns
		V × N	ns	^b^	ns
		W × N	^b^	^b^	^b^
		V × W × N	ns	^b^	^a^

Within a column means followed by different letters are significantly different according to the LSD (0.05). N1, 0 kg N ha^−1^; N2, 30 kg N ha^−1^; N3, 60 kg N ha^−1^, W1,Well-watered; W2, soil water potential was −15 ± 5 kPa; W3, soil water potential was −25 ± 5 kPa; ns, not significant at the 0.05 probability level; ^a^ and ^b^, significant at the 0.05 and 0.01 probability levels, respectively

**Table 4:** Grain yield, head milled rice yield and 2AP content in brown rice of three fragrant rice varieties grown under different water and nitrogen treatments in late season of 2013

Nitrogen	Variety	Water	Grain yield (t ha^− 1^)	Head milled rice yield (t ha^− 1^)	2AP content in brown rice (ug kg^− 1^ DW)
N1	Nongxiang 18	W1	5.98	3.47	113.92
		W2	5.88	3.61	145.43
		W3	5.56	3.44	101.83
	Yungengyou14	W1	5.54	3.35	101.76
		W2	5.51	3.42	112.92
		W3	6.28	3.88	126.73
	Basmati	W1	5.91	3.66	115.21
		W2	5.81	3.53	127.69
		W3	6.04	3.71	102.26
		**Mean**	**5.83 b**	**3.56 b**	**116.42 c**
N2	Nongxiang 18	W1	5.20	3.66	118.77
		W2	5.67	4.03	105.57
		W3	5.98	4.22	139.31
	Yungengyou14	W1	5.83	4.14	155.92
		W2	6.53	4.64	124.86
		W3	6.77	4.81	148.69
	Basmati	W1	5.45	3.80	113.07
		W2	5.55	3.83	128.36
		W3	5.98	4.17	108.85
		**Mean**	**5.88 b**	**4.15 a**	**127.04 b**
N3	Nongxiang 18	W1	6.07	3.91	156.09
		W2	7.12	4.57	142.47
		W3	6.74	4.46	179.48
	Yungengyou14	W1	6.07	3.86	148.13
		W2	7.42	4.85	149.13
		W3	6.45	4.17	199.99
	Basmati	W1	5.48	3.42	128.51
		W2	6.02	3.74	134.47
		W3	6.18	4.10	150.43
		**Mean**	**6.39 a**	**4.12 a**	**154.30 a**
ANOVA		Variety (V)	^b^	^b^	^b^
		Water (W)	^b^	^b^	^b^
		Nitrogen (N)	^a^	^b^	^b^
		V × W	^b^	^b^	^b^
		V × N	^b^	^b^	^b^
		W × N	ns	ns	^b^
		V × W × N	ns	ns	^b^

Within a column means followed by different letters are significantly different according to the LSD (0.05). N1, 0 kg N ha^−1^; N2, 30 kg N ha^−1^; N3, 60 kg N ha^−1^, W1,Well-watered; W2, soil water potential was −15 ± 5 kPa; W3, soil water potential was −25 ± 5 kPa; ns, not significant at the 0.05 probability level; ^a^and ^b^, significant at the 0.05 and 0.01 probability levels, respectively

### Correlation between 2AP content in brown rice and the investigated parameters

The correlation between 2AP content in brown rice and the investigated parameters is shown in Table 5. There was significant positive correlation between 2AP content in brown rice and 2AP content in fresh grain (at 14 d AFH, at 21 d AFH and at MS), leaf (at 7 d AFH and at 14 d AFH) and stem-sheath at 21 d AFH, while, significant negative correlation relationship between 2AP content in brown rice and 2AP content in stem-sheath at FH, 7 d AFH and 14 d AFH was investigated. For proline content, significant positive correlation with 2AP content in brown rice was observed in grain and leaf at 7 d AFH, in stem-sheath at FH, 7 d AFH, 14 d AFH and 21 d AFH and in root at FH and 7 d AFH, the obvious negative correlation with 2AP content in brown rice was found in leaf at 21 d AFH and MS. The significant positive correlation between 2AP content in brown rice was observed at some stages and plant parts. The PDH activity at some stage in leaf, stem-sheath and root showed significant positive correlation relationship with 2AP content in brown rice. Significant positive correlation between 2AP content in brown rice and P5CS activity was observed in some plant parts except in leaf at MS. The DAO activity and OAT activity at some stage in different plant part showed negative correlation relationship with 2AP content in brown rice except for OAT activity in stem-sheath at MS. Overall, the 2AP content in brown rice is positively related to 2AP content, proline content, P5C content, PDH activity and P5CS activity in plant tissue, but negatively with DAO and OAT activity (Table 5).

**Table 5:** Correlation analyses between 2AP content in brown rice and the investigated parameters

Index	FH	7 d AFH	14 d AFH	21 d AFH	MS
2AP					
grain		0.2147 ns	0.2812^a^	0.5429^b^	0.7498^b^
leaf	0.3890^b^	0.6964^b^	0.1884 ns	0.1342 ns	0.2631 ns
Stem-sheath	− 0.6084^b^	− 0.3214^a^	− 0.6450^b^	0.6214^b^	0.2021 ns
Proline					
Grain		0.3932^b^	0.1677 ns	−0.223 ns	− 0.2222 ns
Leaf	0.1045	0.5046^b^	−0.0224	−0.6608^b^	− 0.4014^b^
Stem-sheath	0.4646^b^	0.5116^b^	0.6379^b^	0.4770^b^	−0.1290 ns
Root	0.5538^b^	0.2828^a^	0.1685 ns	0.2453 ns	0.0726 ns
P5C					
Grain		−0.0523 ns	0.2554 ns	0.2870^a^	0.3595^b^
Leaf	0.2937 ns	0.4995^b^	0.4389^b^	0.1976 ns	0.0345 ns
Stem-sheath	0.3997^b^	0.4860^b^	0.4826^b^	0.4271^b^	0.4858^b^
Root	0.4400^b^	0.5269^b^	0.3002^a^	0.4625^b^	0.4085^b^
PDH					
Grain		0.1834 ns	0.0236 ns	0.1666 ns	−0.2187 ns
Leaf	−0.0700 ns	0.3822^b^	0.0587 ns	0.2778^a^	−0.1782 ns
Stem-sheath	0.5317^b^	0.3289^a^	−0.1446 ns	0.4552^b^	0.0974 ns
Root	0.5641^b^	0.1983 ns	0.0040 ns	0.2589 ns	0.2439 ns
P5CS					
Grain		0.1147 ns	0.3975^b^	0.5407^b^	0.3103^a^
Leaf	0.2743 ns	0.3494^b^	0.0739 ns	−0.0577 ns	−0.2724^a^
Stem-sheath	0.3929^b^	0.7658^b^	0.2302 ns	0.5854^b^	0.1644 ns
Root	0.2960^a^	0.5961^b^	0.4466^b^	0.5879^b^	0.1445 ns
DAO					
Grain		−0.0112 ns	−0.1510 ns	−0.5052^b^	− 0.3963^b^
Leaf	−0.0805 ns	− 0.3096^a^	0.1360 ns	0.0167 ns	−0.2337 ns
Stem-sheath	0.0397 ns	−0.3492^b^	0.1060 ns	−0.0642 ns	−0.6041^b^
Root	0.2525 ns	−0.4732^b^	−0.0649 ns	− 0.2147 ns	−0.1632 ns
OAT					
Grain		−0.6194^b^	−0.3658^b^	− 0.4099^b^	−0.1367 ns
Leaf	−0.0063 ns	0.0322 ns	−0.0834 ns	0.0025 ns	0.2044 ns
Stem-sheath	0.4662^b^	0.1237 ns	−0.3382^a^	−0.2943^a^	0.3043^a^
Root	0.2406 ns	−0.0471 ns	−0.4170^b^	− 0.2384 ns	−0.0278 ns

FH, Full heading stage; 7d AFH, 7d after full heading; 14 d AFH, 14d after full heading; 21 d AFH, 21d after full heading; MS, Maturity stage

ns, not significant at the 0.05 probability level; ^a^ and ^b^, significant at the 0.05 and 0.01 probability levels, respectively

### The 2AP accumulation in grains, leaves and stem-sheath

**Fig. 1:**
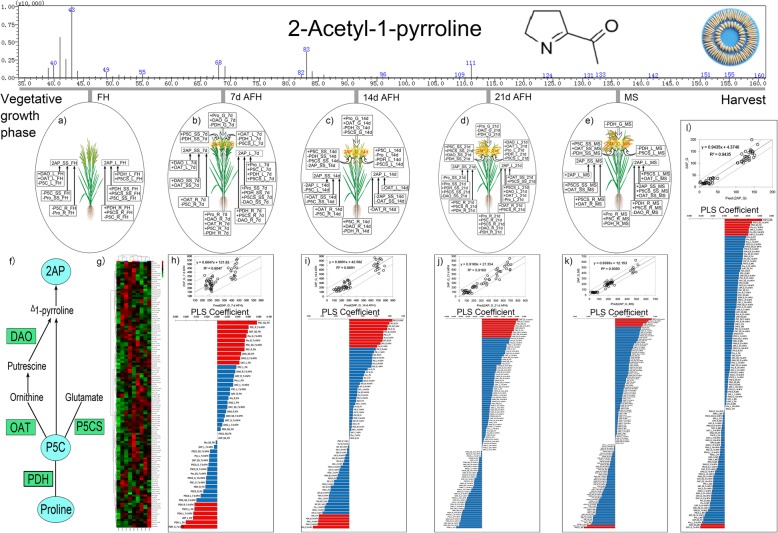
The 2AP accumulation in stem-sheath, leaves and grain at different growth stages. 2AP: 2-acetyl-1-pyrroline, Pro: Proline, PDH: Proline dehydrogenase, P5CS: Pyrroline-5-carboxylic acid synthetase, OAT: Ornithine aminotransferase, P5C: 1-pyrroline-5-carboxylic acid, DAO: Diamine oxidase activity, FH: full heading, AFH: after full heading, MS: maturity stage, L: Leaf, SS: Stem sheath, R: Root, G: Grain

The possible biosynthesis process for 2AP is presented in Fig. 1f. The parameters were detected for different plant tissue at different growth stages. The difference of the investigated parameters in different plant tissues at different growth stages could be classified by the W and N treatments (Fig. 1g).

For plant tissue at FH, the 2AP content in leaf revealed significant positive correlation with PDH and P5CS activity in leaf, stem-sheath root, but significant negative correlation with P5C content in stem-sheath and root. The 2AP content in stem-sheath showed significant positive correlation with DAO and OAT activity in leaf, but revealed significant negative relation with P5C content in leaf, stem-sheath and root and proline content in stem-sheath and root (Fig. 1a).

For plant tissue at 7 d AFH, the 2AP content in grains was significantly positive associated with proline content and DAO activity in grain and root, OAT activity in leaf and root, P5C content in stem-sheath and root, but significant negative related to PDH activity in grain, leaf, stem-sheath and root and P5CS activity in leaf. The 2AP content in leaf was remarkably positive related to proline content in leaf and stem-sheath, PDH and P5CS activity in leaf, stem-sheath and root, P5C content in leaf, but was significantly negative related to DAO activity in stem-sheath and root. The 2AP content in stem-sheath was significantly positive related to OAT activity in leaf, stem-sheath and root, DAO activity in leaf and stem-sheath, but negatively related to P5C content in root (Fig. 1b).

For plant tissue at 14 d AFH, the 2AP content in grains was significantly positive related to P5C content in leaf, stem-sheath and root, proline content and OAT activity in leaf and DAO activity in root. But significant negative related to PDH activity in grain, leaf, stem-sheath and root, P5CS activity in grain, leaf and stem-sheath, 2AP content in stem-sheath and proline content in leaf. The 2AP content in leaf was remarkably negative related to OAT activity in leaf, stem-sheath and root and 2AP content in stem-sheath. The 2AP content in stem-sheath was significantly positive related to OAT activity in stem-sheath and root, but negatively related to P5C content in leaf, stem-sheath and root and 2AP content in leaf (Fig. 1c).

For plant tissue at 21 d AFH, the 2AP content in grains was significantly positive associated with proline content in grain and root, P5C content and P5CS activity in stem-sheath and root, DAO activity in leaf, stem-sheath and root and OAT activity in leaf, but significant negative related to PDH activity in grain, leaf, stem-sheath and root, DAO activity in grain, P5CS activity and proline content in leaf. The 2AP content in leaf was remarkably negative associated with P5CS and OAT activity in leaf, stem-sheath and root and DAO activity in leaf, but showed negative related to proline in leaf. The 2AP content in stem-sheath was significantly positive related to PDH and P5CS activity in stem-sheath and root, proline content in stem-sheath and P5C content in root, but negatively related to proline content in leaf and DAO activity in stem-sheath (Fig. 1d).

For plant tissue at MS, the 2AP content in grains was significantly positive associated with P5C content in stem-sheath and root, OAT activity in stem-sheath and proline content in root. But significant negative related to PDH activity in grain, leaf, stem-sheath and root, P5CS activity in leaf. The 2AP content in leaf was remarkably negative associated with P5CS activity in leaf, stem-sheath and root, OAT activity in leaf and stem-sheath, 2AP content in stem-sheath and PDH activity in root, but showed negative related to DAO activity in stem-sheath and root. The 2AP content in stem-sheath was significantly positive related to OAT activity in leaf, stem-sheath and root, P5CS activity in stem-sheath and root and 2AP content in leaf (Fig. 1e).

It is possible to use each of the parameters in different plant tissue as a response for the experimental data. Data based on the parameters in different plant tissue, a PLS model was established. The correlation graph between the observed and predicted 2AP values has been shown in Fig. 1h-l. The PLS coefficients imply the importance of the parameters with respect to the prediction of the 2AP contents. It was revealed in Fig. 1h, I, j, k and l that the parameters are ranked from the best to the worst fits according to the sizes of their coefficients. There existed difference in parameter contributed to prediction of 2AP content. Nevertheless, the significance for each investigated parameter was included in Fig. 1h-l for the prediction of 2AP content in grain at 7d AFH, 14d AFH, 21d AFH, MS and in brown rice, showed that P5C content, proline content and 2AP content are the significant parameters for 2AP in grains.

Therefore, the 2AP accumulation in different plant tissues related to different biochemical attributes, nevertheless, it could be interpreted by the investigated parameters as illustrated in the analysis correlation and prediction which is shown in Fig. 1.

### Parameters correlated with head milled rice yield and 2AP content

**Fig. 2:**
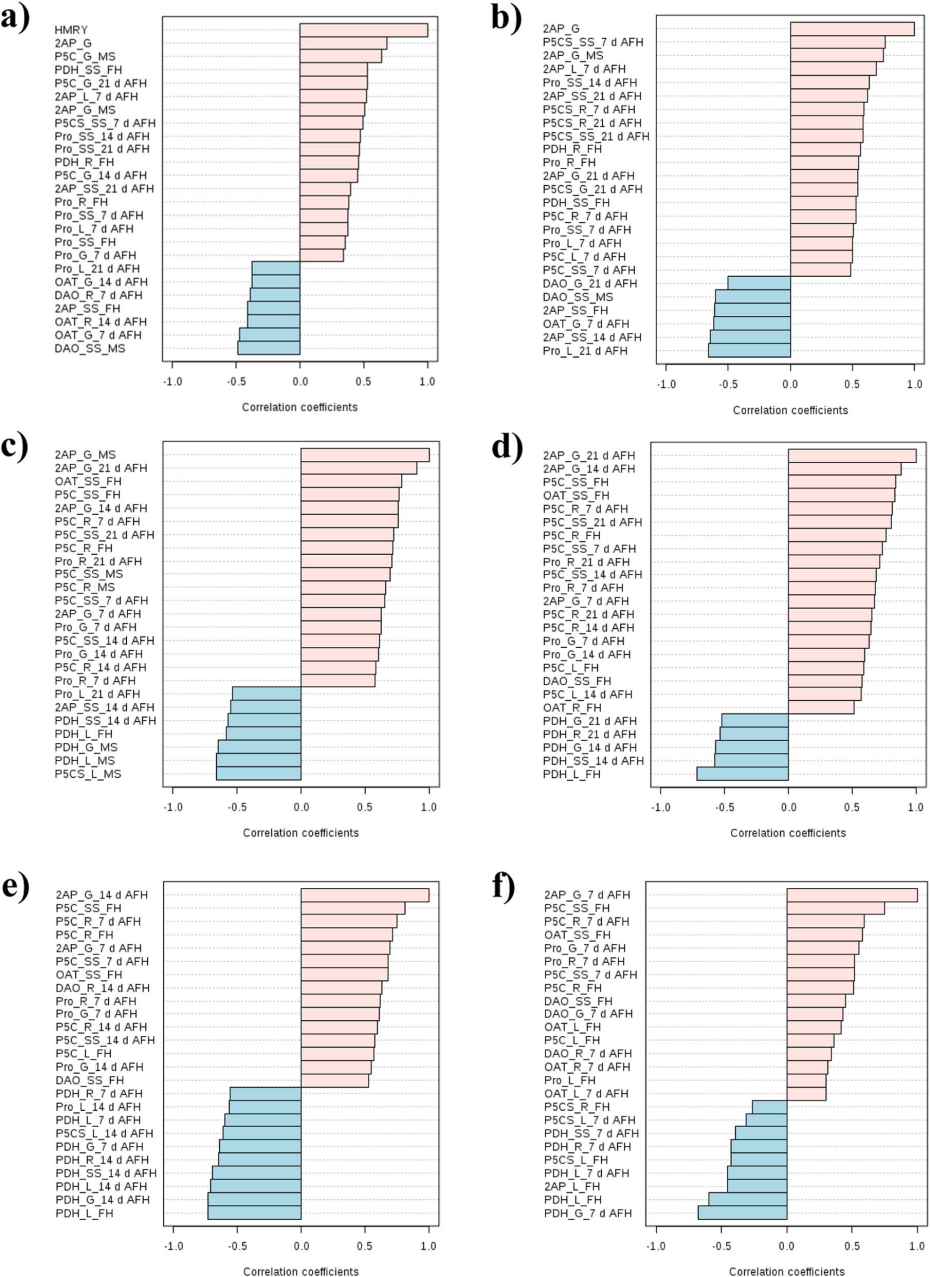
The top 25 parameters correlated with HMRY(**a**), 2AP_G (**b**), 2AP_G_MS(**c**), 2AP_G_21 d AFH (**d**), 2AP_G_14 d AFH (**e**)and 2AP_G_7 d AFH (**f**). HMRY: Head milled rice yield, 2AP_G: 2AP content in brown rice, 2AP_G_MS: 2AP content in grain at maturity stage, 2AP_G_21 d AFH: 2AP content in grain at 21 d AFH, 2AP_G_14 d AFH: 2AP content in grain at 14 d AFH, and 2AP_G_7 d AFH: 2AP content in grain at 7 d AFH

To further reduced the investigated parameters that related to 2AP formation, the top 25 parameters were selected from among all the investigated parameters that strongly correlated with HMRY (Fig. 2a), 2AP_G (Fig. 2b), 2AP_G_MS (Fig. 2c), 2AP_G_21 d AFH (Fig. 2d), 2AP_G_14 d AFH (Fig. 1e) and 2AP_G_7 d AFH (Fig. 2f). For example, 2AP content in brown rice and P5C content in grains at maturity stage (P5C_G_MS) were the top 2 parameters correlated with HMRY. The top 2 parameters correlated with 2AP content in brown rice were P5CS activity in stem-sheath at 7 d AFH (P5CS_SS_7 d AFH), 2AP content in grain at maturity stage (2AP_G_MS). The OAT and DAO activity may contribute to the 2AP content in early grain filling stage (at 7 d AFH, 14 d AFH and 21 d AFH). The groups like P5C content, proline content, the P5CS activity and the PDH activity could be found to contribute to 2AP accumulation during grain filling period (Fig. 2).

### Correlation of the investigated parameters

**Fig. 3:**
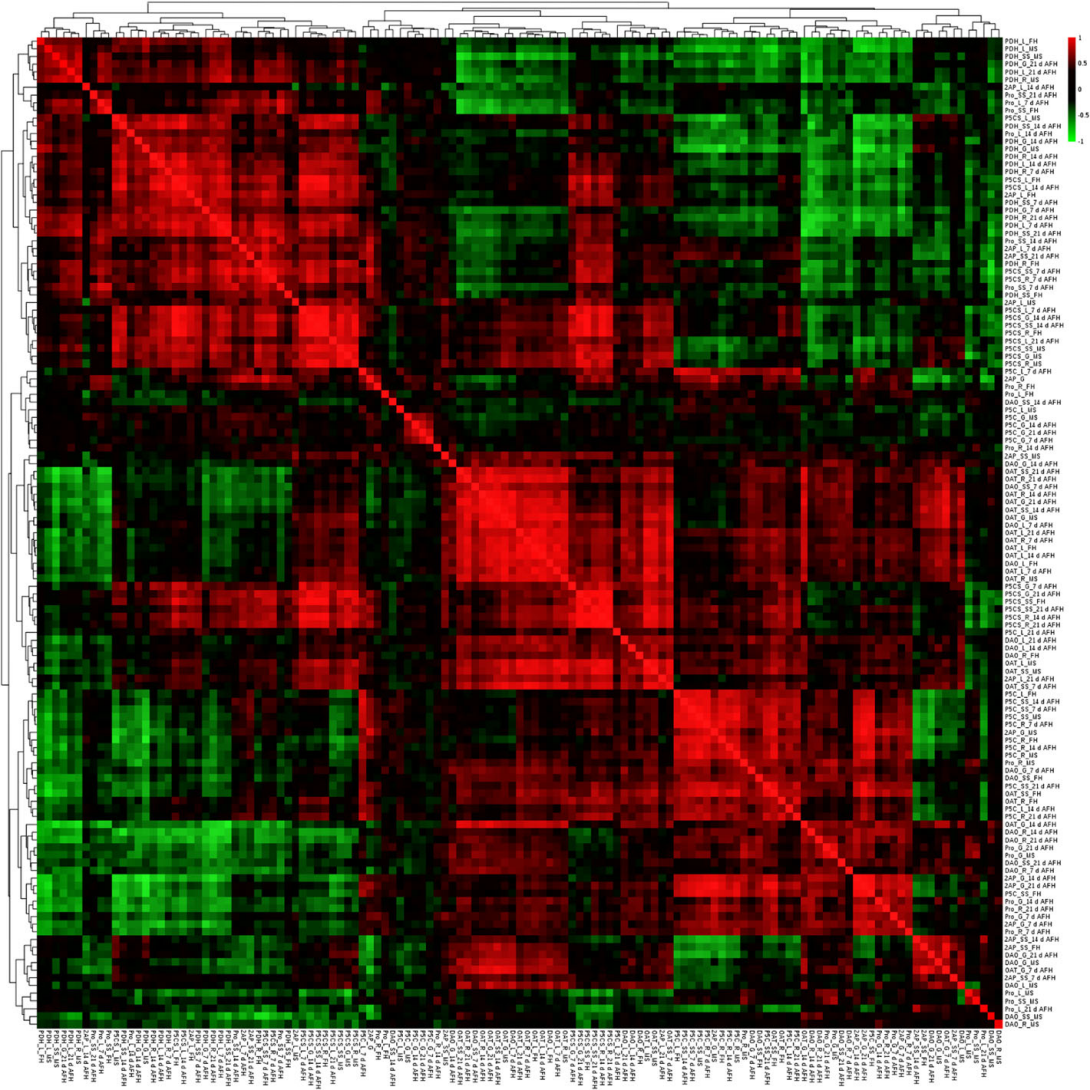
The heatmap for the investigated parameters. 2AP: 2-acetyl-1-pyrroline, Pro: Proline, PDH: Proline dehydrogenase, P5CS: Pyrroline-5-carboxylic acid synthetase, OAT: Ornithine aminotransferase, P5C: 1-pyrroline-5-carboxylic acid, DAO: Diamine oxidase activity, FH: full heading, AFH: after full heading, MS: maturity stage, L: Leaf, SS: Stem sheath, R: Root, G: Grain

To further find out the possible relationships between the investigated parameters the heat map for the investigated parameters was estimated (Fig. 3). It revealed the significant correlation relationship in the groups of PDH activity, P5CS activity; P5CS activity and OAT activity; P5C content and DAO activity; 2AP content, proline content and DAO activity (Fig. 3). It suggested the linking of the physiological attributes that related to 2AP content. The activation of the PDH, P5CS, OAT and DAO balanced the P5C content and proline content and finally affect 2AP content. However, there was still too much redundant data shown in Fig. 3. Therefore a multivariate analysis is further needed.

### PCA and PLS-DA analysis for the investigated parameters

**Fig. 4:**
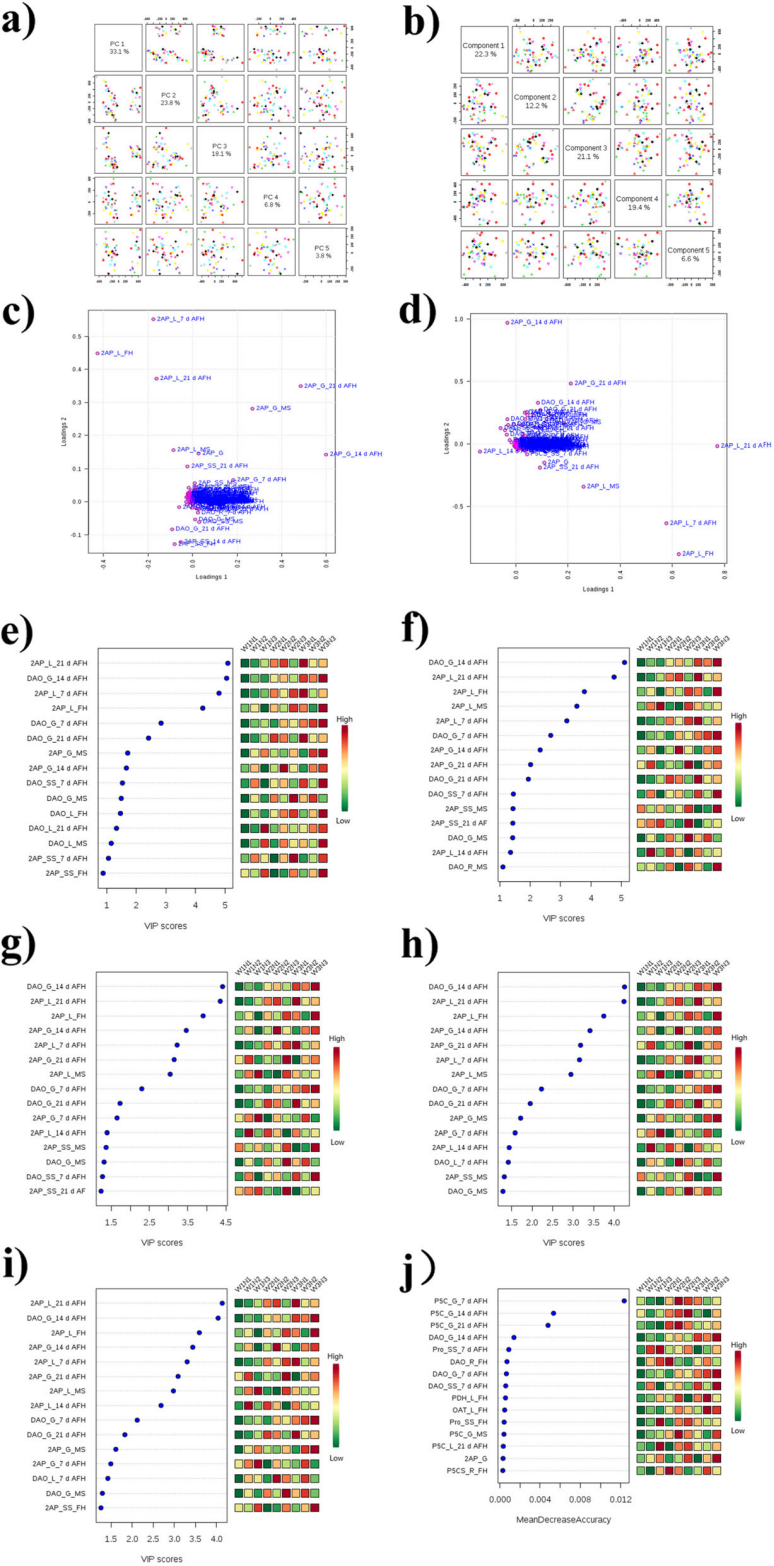
PCA (**a** and **c**) and PLS-DA (**b** and **d**) analysis of the investigated parameters, the important features to the 5 components analysis of PLS-DA for different water and nitrogen treatment (component 1–5: **e**-**i**) and the Parameters ranked by their contributions to classification accuracy (Mean Dicrease Accuracy, **j**)

For further evaluation of the investigated parameters and found out the core parameters, the PCA and PLS-DA analysis were performed. The PCA (Fig. 4a and c) analysis of the investigated parameters revealed that PC1, PC2, PC3, PC4 and PC5 accounted for 33.1%, 23.8%, 18.1%, 6.8% and 3.8%, respectively. The PLS-DA (b and d) analysis indicated that 5 components accounted for the variance with 22.3%, 12.2%, 21.1%, 19.4% and 6.6% for component 1, component 2, component 3, component 4, and component 5, respectively (Fig. 4a and b). The 2AP content in leave and grain were separated from the other variances (Fig. 4c and d). Further, for PCA analysis, the 2AP content in grains and leaves were detected with high loading value for PC1; the 2AP content in grains, leaves and stem sheath were detected with high loading value for PC2. Besides, the DAO activity and 2AP content at some stages in some plant tissues were detected high loading value for PC3, PC4 and PC5 (Additional file 1: Table S1). The further analysis of the important features to the 5 components analysis of PLS-DA and the parameters ranked by their contributions to classification accuracy under water and nitrogen treatment was shown in Fig. 5. A detail PLS-DA loading of the parameters was calculated (Additional file 2: Table S2). The 2AP content and DAO activity were the key parameters that associated with W and N treatments in the 5 components established by PLS-DA analysis (Fig. 5e-i). Moreover, the analysis of the parameters ranked by their contributions to classification accuracy revealed that the top 3 parameters were P5C content in grain at 7 d AFH, 14 d AFH and 21 d AFH for different W and N treatments (Fig. 5j). Therefore, the 2AP content and P5C content in different plant tissues accounted for most of the contributions under W and N treatment of the three fragrant rice varieties.

## Discussion

The 2-acetyl-1-pyrroline (2AP) is a major aromatic compound for aroma of fragrant rice (Buttery et al., 1983; Magnus et al., 2002, Bryant and McClung, 2011; Grimm et al., 2011; Wakte et al., 2017; Routray and Rayaguru, 2017). The effect of the environmental factors such as the temperature, light and nutrition, and the cultivation practices such as irrigation, fertilization, and plant regulators on 2AP formation during rice growth period have been evaluated in previous studies (Bhattacharjee et al., 2002; Champagne, 2008; Goufo et al., 2010; Poonlaphdecha et al., 2012; Yang et al., 2012; Mo et al., 2015, 2016; Li et al., 2016, b). Those results suggested that it is feasible to regulate the 2AP content in fragrant rice with moderate irrigation and nitrogen application (Yoshihashi et al., 2002; Itani et al., 2004; Tian et al., 2010; Wang et al., 2013; Bao et al., 2018; Mo et al., 2018). It have been reported that water and nitrogen management at tillering stage can regulate 2AP accumulation (Li, Tang, et al., 2014; Ren et al., 2017). In this study, applied nitrogen fertilizer 30 kg N ha^− 1^ (N2) and 60 kg N ha^− 1^ (N3) and at booting stage increased 2AP content in brown rice, grain yield and head milled rice yield across three varieties and three water irrigation treatments. The soil water potential of − 25 ± 5 kPa (W3) treatment at booting stage improved grain yield, head milled rice yield and 2AP content in fragrant rice under different nitrogen condition. Strong water and nitrogen interaction effect on 2AP content in brown rice was detected and 60 kg N ha^− 1^ plus − 25 ± 5 kPa treatment yielded strong 2AP content in brown rice (Tables 3 and 4). Moreover, the 2AP content, proline content and P5C content as well as the PDH activity and P5CS activity were investigated as the important parameters that positively related to the 2AP content in brown rice under different W and N treatments, while the DAO and OAT activity was detected as negatively related to the 2AP content in brown rice (Table 5).

In general, 2AP have been detected in different plant tissues of fragrant rice plant such as grain, stem sheath and leaf but root (Buttery et al., 1983; Maraval et al., 2010). In this study, the 2AP content in brown rice, grains, leaves and stem sheath was also detected (Tables 3 and 4, Fig. 1). The dynamic of the 2AP and its related biochemical attributes under W and N treatments at booting stage were investigated, the correlations between 2AP and the biochemical parameters and prediction of 2AP content by the biochemical attributes from different plant tissues and growth stages were assessed (Fig. 1). The difference in 2AP accumulation in different plant part indicated the difference of 2AP formation physiological basis in different plant tissues (Buttery et al., 1983; Maraval et al., 2010). In this study, the relationship between the 2AP content and its related biochemical parameters in different plant tissues were revealed (Fig. 1a-e). Besides, the important parameters that related to head milled rice yield, 2AP content in brown rice, 2AP in grains at MS, 21 d AFH, 14 d AFH and 7 d AFH have been detected and the result suggested that the groups like P5C content, proline content, the P5CS activity and the PDH activity could be found to contribute to 2AP accumulation during grain filling period (Fig. 2). Moreover, it could be fully predicted of 2AP by using the related biochemical parameters (Fig. 1h-l).

The precursors (proline, ornithine, glutamic acid, P5C, pyrrole and 1-pyrroline) and the enzymes (PDH, P5CS, OAT and DAO) have been reported to be related to 2AP formation (Seitz et al., 1993; Huang et al., 2008; Chen et al., 2008; Bradbury et al., 2008; Sakthivel et al., 2009; Fitzgerald et al., 2009, 2010; Wakte et al., 2011; Kaikavoosi et al., 2015; Daygon et al., 2017; Ghosh and Roychoudhury, 2018). However, the precursors and the enzymes involved in the 2AP accumulation in different plant tissues and their dynamics were different from different plant tissues (Fig. 1). Previous studies have revealed variations in the correlation between the 2AP content and the biochemical attributes under different fertilization treatment and varieties (Mo et al., 2016, b; Li et al., 2016, b; Ghosh and Roychoudhury, 2018). The study of Li et al. (2016, b) revealed significant and positive correlation between 2AP and P5C content in fragrant rice. Mo et al. (2017) also demonstrated PDH activity and proline content were connected to the 2AP formation and accumulation. Proline is a precursor of 2AP in fragrant rice and higher proline content yield strong 2AP content (Seitz et al., 1993; Huang et al., 2008; Poonlaphdecha et al., 2012). Study also reported that P5C is significantly positive correlated to 2AP in grains (Mo et al., 2016, b). In our study, the 2AP content in leave and grain were separated from the other parameters (Fig. 4c and d). The 2AP content was the important parameter under water and nitrogen treatments for PCA analysis (Supplement S1). Besides, the important features accounted for the 5 components of PLS-DA analysis for water and nitrogen treatment was the 2AP content and DAO activity (Fig. 4e-i). Further, the analysis of the parameters ranked by their contributions to classification accuracy revealed that the top 3 parameters were P5C content in grain at 7 d AFH, 14 d AFH and 21 d AFH under water and nitrogen treatments (Fig. 4j).

Over all, these results suggested the 2AP content, P5C content and DAO activity during grain filling periods was highly related to the 2AP content in brown rice under different water and nitrogen treatments.

## Conclusion

Application of N fertilizer 30 and 60 kg N ha^− 1^ at booting stage increased 2AP content in brown rice, grain yield and head milled rice yield across three rice varieties and three water treatments. The soil water potential of − 25 ± 5 kPa (W3) treatment at booting stage improved grain yield, head milled rice yield and 2AP content in fragrant rice under different nitrogen condition. Moreover, strong W and N interactions on 2AP content in brown rice was detected and 60 kg N ha^− 1^ plus − 25 ± 5 kPa treatment yielded strong 2AP content in brown rice. The 2AP content, P5C content and DAO activity during grain filling periods was highly related to the 2AP content in brown rice. Overall, present study revealed that the 60 kg N ha^− 1^ plus − 25 ± 5 kPa treatment showed the positive effect on yield and aroma in fragrant rice, suggested that W and N management at booting stage could improve grain yield and aroma biosynthesis in fragrant rice. Further study to evaluate the metabolic and molecular basis of 2AP accumulation in fragrant rice is needed.

## Additional files

Additional file 1: **Table S1.** PCA_loadings (XLSX 19 kb)

Additional file 2: **Table S2.** PLS-DA_loadings (XLSX 19 kb)

## Data Availability

All data supporting the conclusions of this manuscript are provided within the manuscript.

## Abbreviations

2AP: 2-acetyl-1-pyrroline
AFH: After full heading
DAO: Diamine oxidase activity
FH: Full heading
G: Grain
HMRY: Head milled rice yield
L: Leaf
MS: Maturity stage
OAT: Ornithine aminotransferase
P5C: 1-pyrroline-5-carboxylic acid
P5CS: Pyrroline-5-carboxylic acid synthetase
PDH: Proline dehydrogenase
Pro: Proline
R: Root
SS: Stem sheath
