# Correction to: Nitrogen application and different water regimes at booting stage improved yield and 2-acetyl-1-pyrroline (2AP) formation in fragrant rice

**DOI:** 10.1186/s12284-019-0335-5

**Published:** 2019-10-31

**Authors:** Zhaowen Mo, Yanhong Li, Jun Nie, Longxin He, Shenggang Pan, Meiyang Duan, Hua Tian, Lizhong Xiao, Keyou Zhong, Xiangru Tang

**Affiliations:** 1 Department of Crop Science and Technology, College of Agriculture, South China Agricultural University, Guangzhou, 510642 Guangdong China; 2 Scientific Observing and Experimental Station of Crop Cultivation in South China, Ministry of Agriculture.P. R. China, Guangzhou, 510642 China; 3 Agro-innovative Demonstration Base Guangdong Academy of Agricultural Sciences, Guangzhou, 510642 China

**Correction to: Rice (2019) 12:74**

**https://doi.org/10.1186/s12284-019-0328-4**

It was highlighted that the original article (Mo et al. 2019) contained an error in Fig. 1f which revealed the biosynthesis pathway of 2AP. This Correction article shows the correct Fig. 1 and incorrect Fig. 1. The original article has been updated.

Correct Fig. 1

Incorrect Fig. 1
